# Frequency of Parkinson’s Disease Genes and Role of *PARK2* in Amyotrophic Lateral Sclerosis: An NGS Study

**DOI:** 10.3390/genes13081306

**Published:** 2022-07-22

**Authors:** Veria Vacchiano, Anna Bartoletti-Stella, Giovanni Rizzo, Patrizia Avoni, Piero Parchi, Fabrizio Salvi, Rocco Liguori, Sabina Capellari

**Affiliations:** 1 IRCCS Istituto Delle Scienze Neurologiche di Bologna, Bellaria Hospital, 40139 Bologna, Italy; veriavacchiano@gmail.com (V.V.); g.rizzo@unibo.it (G.R.); patrizia.avoni@unibo.it (P.A.); piero.parchi@unibo.it (P.P.); fabrizio.salvi@gmail.com (F.S.); rocco.liguori@unibo.it (R.L.); 2 Dipartimento di Scienze Biomediche e Neuromotorie (DIBINEM), Università di Bologna, 40123 Bologna, Italy; 3 Department of Experimental Diagnostic and Specialty Medicine (DIMES), University of Bologna, 40138 Bologna, Italy; anna.bartoletti2@unibo.it

**Keywords:** amyotrophic lateral sclerosis, Parkinson’s disease, PD-related genes, GBA, neurodegenerative diseases, Alzheimer’s disease

## Abstract

Amyotrophic lateral sclerosis (ALS) and Alzheimer’s disease (AD) patients show a higher prevalence of Lewy body disease than the general population. Additionally, parkinsonian features were found in about 30% of ALS patients. We aimed to explore the frequency of Parkinson’s disease (PD)-causative genes in ALS patients, compared to AD and healthy controls (HCs). We used next-generation sequencing multigene panels by analyzing *SNCA*, *LRRK2*, *PINK1*, *PARK2*, *PARK7*, *SYNJ1*, *CHCHD2*, *PLA2G6*, *GCH1*, *ATP13A2*, *DNAJC6* and *FBXO* genes. *GBA* gene, a risk factor for PD, was also analyzed. In total, 130 ALS and 100 AD patients were investigated. PD-related genes were found to be altered in 26.2% of ALS, 20% of AD patients and 19.2% of HCs. Autosomal recessive genes were significantly more involved in ALS as compared to AD and HCs (*p* = 0.021). *PARK2* variants were more frequent in ALS than in AD and HCs, although not significantly. However, the p.Arg402Cys variant was increased in ALS than in HCs (*p* = 0.025). This finding is consistent with current literature, as parkin levels were found to be decreased in ALS animal models and patients. Our results confirm the possible role of PD-related genes as risk modifier in ALS pathogenesis.

## 1. Introduction

Amyotrophic lateral sclerosis (ALS) is a fatal neurodegenerative disease characterized by the degeneration of both upper and lower motor neurons. Although classically considered a “pure” motor disease, up to 50% of cases develop extra-motor features such as cognitive decline, configuring the diagnosis of frontotemporal dementia (FTD) in 10–15% of patients [1]. In addition, epidemiological studies demonstrated that offspring of Parkinson’s disease (PD) patients exhibit an increased risk for developing ALS [2], and ALS patients may develop parkinsonian features in up to 30% of cases [3]. Moreover, a recent study showed a higher prevalence of Lewy body disease (LBD) in ALS patients than in the general population [4]. 

A genetic link between ALS and PD pathogenesis has already been established by the largest genome-wide association study (GWAS) on ALS patients, underlying a locus-specific pattern of shared genetic risk across several neurodegenerative diseases [5]. 

Of note, the most frequent gene associated with ALS, the *C9Orf72* gene, can manifest with typical or atypical parkinsonism [6,7], and some pleiotropic genetic disorders, such as multisystemic proteinopathy, can present with both ALS and parkinsonian features [8]. 

Overlapping of different degenerative diseases is a frequent phenomenon, for example, in patients with Alzheimer’s disease (AD), where around 50–60% of sporadic and genetic patients, carrying pathogenic mutations in *APP*, *PSEN1*, or *PSEN2* genes, manifest widespread α-synuclein pathology [9,10].

Around 5–10% of PD is defined as monogenic disorders. Autosomal dominant forms can be caused by very rare highly penetrant mutations in the α-synuclein (*SNCA*), responsible for about 2% of familial PD [11,12,13], or by the most frequent *LRRK2* gene, mainly causing late-onset PD with a milder phenotype. Autosomal recessive forms are caused by mutations in the *PARK2*, *PINK1*, and *PARK* genes, which approximately share a similar phenotype, characterized by early onset parkinsonism responsive to levodopa [11,12,13]. Besides the most frequent genes, several new loci have been recently associated with PD pathogenesis, even though their molecular function has not been fully defined [12,13]. In addition, heterozygous *GBA* (OMIM *606463) mutations increase the risk for PD [14] and LBD [15]. Of interest, rare variants in the *GBA* gene have also been associated with cognitive decline in ALS patients [16], although their frequency was not found to be increased in ALS patients compared to healthy controls. 

To better define this topic, we performed a systematic screening of causal genes for PD and the *GBA* gene in a cohort of ALS patients. We also extended the genetic analysis to a cohort of AD patients, to compare results from an ALS cohort with those from another neurodegenerative disease known to often have an associated α-synuclein pathology. 

## 2. Materials and Methods

We analyzed known PD-causing genes, i.e., *SNCA*, *LRRK2*, *PINK1*, *PARK2*, *PARK7*, *SYNJ1*, *CHCHD2*, *PLA2G6*, *GCH1*, *ATP13A2*, *DNAJC6* and *FBXO7* (Supplementary Materials, Table S1) by next-generation sequencing (NGS) multigene panels [17]. *GBA* gene, a risk factor for PD, was included in the genetic analysis. 

Briefly, genomic DNA from peripheral blood was isolated using the Maxwell 16 extractor (Promega, Madison, WI, USA) and quantified using the Quantus Fluorometer (Promega). Analyses of coding and splice site regions of genes of interest were performed by multigene panels, by using either one of the following panels: amplicon-based Illumina panel (TruSeq Custom amplicon, Illumina, San Diego, CA, USA) and probe-based Illumina panel (Truseq Neurodegeneration Illumina). Sequencing was performed on a MiSeq or NextSeq 500 sequencer using the Illumina V2 reagent kit, with 2 × 150 bp paired end read cycles. Sequencing data were analyzed with an in-house bioinformatic pipeline. Variant filtration and depth of coverage analysis were performed using Genome Analysis Toolkit (GATK) v4 [18].

Variant selection was performed with BaseSpace Variant interpreter (Illumina, San Diego, CA, USA), following these criteria: sequence read depth at least 10× and for heterozygous variants, an allelic balance value in the range of 0.35 and 0.70, and minor allele frequency (MAF) < 1% in the Genome Aggregation Database (GnomAD) [19]. Variants were classified according to the American College of Medical Genetics and Genomics (ACMG) guidelines for the interpretation of sequence variants provided by Franklin-Genoox (franklin.genoox.com, accessed on 26 May 2022). For this study, we considered variants classified as pathogenic, likely pathogenic or of uncertain significance (VUS). As reported in Mighton et al., 2022 [20], we considered synonymous variants which are not predicted to impact splicing (BP7) as likely benign. In addition, we defined as likely benign missense VUS variants that met the BP6 criterion (Reputable source recently reports variant as benign, but the evidence is not available to the laboratory to perform an independent evaluation) [21].

All ALS patients diagnosed according to the Revised El Escorial criteria [22] were already screened for mutations in the major ALS genes: *SOD1*, *FUS*, *TARDBP*, and *C9orf72* genes, as previously reported [19]. Clinical data including gender, age at onset, ALS phenotype [23], and disease duration (from clinical disease onset to death) were collected. 

A cohort of AD patients diagnosed according to the International Working Group 2 criteria [24] was also analyzed. 

As healthy controls, we used the recently published whole-exome sequencing data set composed of 1686 healthy Italian individuals [25]. In detail, we selected variants with an allele frequency < 1% and a reported effect that included: missense, synonymous, structural interaction variant, splice variant frameshift variant. Variants were classified as previously reported for the ALS cohort.

The study was conducted according to the revised Declaration of Helsinki and Good Clinical Practice guidelines and approved by the local ethics committee “Area Vasta Emilia Centro” (approval code: CE-AVEC—17151—17152; approval date: 29 March 2021). Informed consent was given by participants for their participation in the study and for the publication of data.

Statistical analysis was performed using IBM SPSS Statistics version 25 (IBM, Armonk, NY, USA). Quantitative continuous variables were described as median and interquartile range or as mean and standard deviation according to their distribution. Categorical variables were expressed as counts and percentages. For continuous variables, the Mann–Whitney U test was used to test differences between two groups. The chi-squared test was adopted for categorical variables. For statistical tests, *p* < 0.05 was considered significant.

## 3. Results

In total, 130 ALS and 100 AD patients were included in the study. Nineteen ALS patients (14.6%) carried a pathogenic variant in one of the four major ALS genes (Supplementary Materials, Table S2), while no pathogenic variants in causative AD genes were found in AD patients. Distribution of PD-related gene variants is detailed in Table 1.

31 ALS patients (23.8%) and 17 AD patients (17%) carried at least one variant in a PD-related gene. In detail, 34 PD-related variants were found in ALS (26.2%) compared to 20 AD (20%) and 323 healthy controls (19.2%). The frequency of PD-related variants resulted slightly higher in ALS than in AD and healthy controls (*p* = 0.15). Variants in PD-related autosomal dominant genes were found in 2 [1.5%] ALS, 2 [2%] AD patients, and 72 [4.3%] healthy controls, with no significant differences (*p* = 0.18). No patients carried *SNCA* variants in either degenerative cohort. The frequency of PD-related variants in autosomal recessive genes was significantly higher in patients with ALS as compared to AD and healthy controls (31 [23.8%] vs. 18 [18%] vs. 251 [14.9%], *p* = 0.021 adjusted for Bonferroni). Variants in the *PARK2* gene were more frequent in ALS patients than in healthy controls, although not significantly (6.2% vs. 4%, *p* = 0.47), while the frequency in AD and the healthy population was quite similar. However, regarding the *PARK2* gene, we observed that 6 ALS patients carried the same variant, p.Arg402Cys. This variant was classified as VUS despite being found in a homozygous state in the GnomAD population. This variant was not identified in our AD-cohort, and was found with an allele frequency of 0.0089 in the Italian population used as a control (NIG-database). Despite the “not very low” frequency of this variant, the Chi-square test suggested enrichment in our ALS cohort (4.6 *vs* 1.8, *p* = 0.025).

*GBA* variants were more frequent in AD than in ALS patients, but both frequencies were quite similar to those in healthy controls [16]. Three ALS patients (ALS-2, ALS-25 and ALS-98) carried more than one PD-related variant (Table 2), while another patient (ALS-102) carried two *GBA* variants (c.1093G>A, c.882T>G). 

Clinical features of ALS patients carrying variants in PD-related genes are detailed in Table 2, Table 3 and Table 4. The classification of the variants found in ALS and AD cohorts is detailed in the Supplementary Materials (Tables S3–S5).

After excluding the 19 ALS patients carrying a mutation in one of the four major ALS genes, we compared the clinical features of ALS patients carrying at least one variant in PD-related genes with the remaining ALS patients (Table 5). 

Age at onset and disease duration did not significantly differ between the two groups (*p* = 0.53 and 0.93, respectively). Similarly, we did not find any significant difference between the two groups regarding the family history distribution (ALS/dementia and/or parkinsonism) (*p* = 0.49) or the ALS phenotypic variant (*p* = 0.17).

## 4. Discussion

In this study, we systematically explored the frequency of variants in PD-associated causative genes and frequency of the *GBA* gene in two cohorts of patients with neurodegenerative disease known to harbor a higher prevalence of Lewy body disease (LBD) than the general population and compared them with the genes’ frequencies in Italian controls [25]. We found a significant increase of variants in autosomal recessive PD-related genes in ALS than in AD and healthy controls. 

Variants apparently play no role in modifying the phenotype since we failed to find different clinical features among ALS patients carrying at least one PD-related gene variant compared to patients without them. Although possibly affected by the relatively small sample size, our results are in line with the largest GWAS study to date [5], showing that even some confirmed genetic risk factors for ALS have a limited effect on survival and age of onset, highlighting that knowledge of factors underlying the high variability of phenotypes of ALS is still poor. 

Regarding the most frequent autosomal dominant genes causing PD, we did not find any *SNCA* variant in both ALS and AD cohorts. Of note, previous studies [26,27] reported that polymorphisms in the *SNCA* gene, shown to affect the risk of developing PD, were not increased in ALS, and did not influence ALS clinical features.

The *LRRK2* gene, encoding the dardarin protein, has been reported in familial PD [28]. In our study, we found two *LRRK2* variants in ALS and two variants in AD patients (Table 2), but the gene frequency was not different compared to healthy controls. Interestingly, an *LRRK2* mutation (5096A>G) was first found in a German-Canadian family, with a complex clinical spectrum of parkinsonism, dementia, and lower motor neuron signs [29]. Subsequently, another study failed to find validated pathogenic *LRRK2* variants (i.e., R1441G=C=H, Y1699C, I2012T, G2019S, and I2020T) in 54 ALS patients [30]. Conversely, Ciani M et al. [31] found a pathogenic *LRRK2* variant (c.T4937C, p.M1646T) in a patient with sporadic FTD. Interestingly, a correlation between specific *LRRK2* variants and plasma progranulin levels was highlighted by Caesar et al. [32], suggesting that *LRRK2* mutations could be implicated in the ALS-FTD spectrum, also based on their role in altering one or more specific pathways, including autophagy, immune response, neurite outgrowth, and vesicle trafficking [33]. However, the inconclusive results from our study and several studies did not allow us to draw definitive conclusions about its role in ALS pathogenesis.

Homozygous mutations in *PARK2* and *PINK1* are the most known causes of autosomal recessive early onset parkinsonism [13,34]. Furthermore, single heterozygous *PARK2* or *PINK1* variants are higher in PD patients than controls and have been associated with subclinical parkinsonism [35]. *PARK2* and *PINK1* are essential regulators of mitophagy, one of the autophagy pathways for selective removal of dysfunctional mitochondria. Parkin depletion has been classically associated with recessive early onset PD due to loss-of-function mutations in the *PARK2* gene, whereas in sporadic PD, the protein is abundant, but its function can be inactivated by stress-related modifications. 

We found a higher frequency of *PARK2* variants in ALS as compared to healthy controls and AD patients (although not significant), and one in ALS as compared to two *PINK1* variants in AD. Interestingly, we found a high frequency of the p.Arg402Cys variant in the *PARK2* gene in the ALS cohort. This variant is classified as a variant of uncertain significance in ClinVar and has been also recently found to be increased in a FTD cohort [36], where the authors speculated the possible role of the *PARK2* gene in the pathogenesis of FTD, highlighting the possible overlap across neurodegenerative diseases. 

Intriguingly, recent research has shown that parkin depletion is not limited to PD but is also observed in other neurodegenerative diseases, especially those characterized by TDP-43 proteinopathies, such as ALS and frontotemporal lobar degeneration [35]. In particular, decreased levels of parkin have been found in mouse adult brain, stem-cell-derived human neurons, human fibroblasts, and motor neurons from sporadic ALS patients [35]. Furthermore, in motor neurons derived from sporadic ALS patients, a correlation has been established between decreased parkin and the presence of TDP-43 aggregates [37]. Although loss of function of TDP-43 has been directly correlated with decreased parkin expression, the exact significance of this phenomenon and its consequences remains elusive.

On the other hand, altered expression of *PINK1*, both at mRNA and protein levels, has been identified in ALS patients’ muscle [38]. 

Finally, in our study, *GBA* variants were not differently distributed between ALS and AD patients. 

The *GBA* gene encodes the lysosomal enzyme β-glucocerebrosidase, which is involved in the breakdown of the glucocerebroside, a complex component of cellular membrane, into glucose and ceramide [34]. Homozygous mutations in *GBA* gene are recognized as causative in patients affected by Gaucher’s disease [39], but they were also identified as important risk factors in PD and LBD patients [40,41]. Moreover, heterozygous *GBA* carrier PD patients showed an increased risk of developing the disease, an earlier onset age, and faster progression. In addition, more frequent cognitive decline and alterations in executive function and language processing were observed in patients carrying these variants [42]. Interestingly, pathogenic variants in *GBA* have also been found in FTD [31], and ALS patients show in the latter a more compromised cognitive profile [16]. Our results are in line with previous studies [16,43], confirming that the frequency of *GBA* variants in the ALS cohort is similar to that of European population controls in the gnomAD database. 

Our study has some limitations. The lack of systematic neuropsychological assessment did not allow us to explore the association between *GBA* variants or PD-related genes and the cognitive profile in ALS patients. Furthermore, the relatively small samples of patients did not allow us to draw definitive conclusions on the association of genetic variants with clinical features. To the best of our knowledge, this is the first study that systematically explored an extensive battery of PD-related genes in ALS patients. We showed an increase of variants in autosomal recessive PD-related genes in ALS, confirming a possible pathogenic role of other neurodegenerative diseases’ related genes as risk modifiers in ALS. In particular, the role of the *PARK2* gene, a variant of which, the p.Arg402Cys, has been shown to be enriched as much in our cohort of patients with ALS as in a cohort of patients with FTD previously, is intriguing.

Further studies are needed to confirm our results and to better understand the pathogenic role of the PD-related genes in the ALS pathogenic mechanism.

## Figures and Tables

**Table 1:** Distribution of PD-related gene variants in ALS, AD patients, and healthy controls.

Gene	ALS (130)	AD (100)	HC (1686)
*LRRK2*	2 (1.5%)	2 (2%)	62 (3.7%)
*ATP13A2*	4 (3.1%)	4 (4%)	45 (2.7%)
*DNAJC6*	3 (2.3%)	1 (1%)	20 (1.2%)
*FBXO7*	2 (1.5%)	1 (1%)	20 (1.2%)
*PINK1*	5 (3.8%)	1 (1%)	23 (1.4%)
*PARK7*	1 (0.8%)	0	12 (0.7%)
*GCH1*	1 (0.8%)	0	10 (0.6%)
*PARK2*	8 (6.2%)	4 (4%)	70 (4.2%)
*SYNJ1*	6 (4.6%)	4 (4%)	29 (1.7%)
*CHCHD2*	1 (0.8%)	1 (1%)	7 (0.4%)
*PLA2G6*	1 (0.8%)	2 (2%)	25 (1.5)
*GBA*	2 (1.5%)	3 (3%)	2.2% [16]

Key: ALS, amyotrophic lateral sclerosis; AD, Alzheimer’s disease; HC: healthy controls.

**Table 2:** Clinical features of ALS patients carrying autosomal dominant genes.

Patient	Variant	Classification (Franklin ACMG Classification)	Sex	Family History	Onset Age	Phenotype	DD (Months)
**ALS-25**	***LRRK2*** **c.7470A>C p.Gln2490His**	VUS	**F**	**None**	**69**	**Classic**	**73**
ALS-29	*LRRK2* c.7315C>A p.Leu2439Ile	VUS	M	None	72	PUMN	60

Key: ALS, amyotrophic lateral sclerosis; DD, disease duration; F, female; *LRRK2*, LEUCINE-RICH REPEAT KINASE 2; M, male; PUMN, predominant upper motor neuron; VUS, variant of uncertain significance. In bold: patients carrying more than one variant.

**Table 3:** Clinical features of ALS patients carrying autosomal recessive genes.

Patient	Variant	Classification (Franklin ACMG Classification)	Sex	Family History	Onset Age	Phenotype	DD (Months)
ALS-24	*ATP13A2 c.2836A>T* p.Ile946Phe	VUS	M	Dementia and PD	47	PLMN	47
ALS-40	*ATP13A2* c.1382delC p.Ala461ValfsTer5	Likely pathogenic	F	None	87	Classic	24
ALS-46	*ATP13A2* c.3059A>G p.Tyr1020Cys	VUS	F	None	51	Classic	113
ALS-91	*ATP13A2* c.2947C>T p.Pro983Ser	VUS	F	None	52	Classic	36
**ALS-2**	***CHCHD2*** **c.101C>T p.Pro34Leu**	**VUS**	**M**	**None**	**63**	**Classic**	**50**
**ALS-2**	***DNAJC6* c.1112A>C p.Lys371Thr**	**VUS**	**M**	**None**	**63**	**Classic**	**50**
ALS-27	*DNAJC6* c.829G>A p.Ala277Thr	VUS	M	None	49	Classic	73
ALS-81	*DNAJC6* c.2048C>T p.Thr683Met	VUS	M	None	65	Classic	24
ALS-60	*FBXO7* c.301A>C p.Asn101His	VUS	M	None	71	Classic	33
**ALS-98**	***FBXO7* c.1538G>A p.Arg513Gln**	**VUS**	**F**	**Dementia**	**62**	**Classic**	**119**
ALS-15	*PINK1* c.1342G>A p.Gly448Arg	VUS	F	None	56	PUMN	Unknown
ALS-18	*PINK1* c.434C>T p.Thr145Met	VUS	M	None	64	PLMN	154
ALS-53	*PINK1* c.1609G>A p.Ala537Thr	VUS	M	Dementia	54	PUMN	51
**ALS-98**	***PINK1* c.587C>T p.Pro196Leu**	**VUS**	**F**	**Dementia**	**62**	**Classic**	**119**
ALS-106	*PINK1* c.587C>T p.Pro196Leu	VUS	F	Dementia	74	Classic	22
ALS-103	*PARK7* c.535G>A p.Ala179Thr	VUS	M	None	38	Classic	Unknown
ALS-28	*GCH1* c.671A>G p.Lys224Arg	Likely pathogenic	F	None	73	Classic	44
ALS-12	*PARK2* c.1204C>T p.Arg402Cys	VUS	M	None	66	PLMN	21
**ALS-25**	***PARK2*** **c.1204C>T p.Arg402Cys**	**VUS**	**F**	**None**	**69**	**Classic**	**73**
ALS-64	*PARK2* c.1204C>T p.Arg402Cys	VUS	M	None	39	PLMN	Unknown
ALS-80	*PARK2* c.1204C>T p.Arg402Cys	VUS	M	None	55	PUMN	24
ALS-113	*PARK2* c.1204C>T p.Arg402Cys	VUS	M	Dementia	40	PLMN	Unknown
ALS-126	*PARK2* c.1204C>T p.Arg402Cys	VUS	M	None	36	Classic	10
ALS-100	*PARK2* c.701G>A p.Arg234Gln	VUS	F	Dementia	57	Classic	41
ALS-129	*PARK2* c.436T>C p.Phe146Leu	VUS	M	None	63	Classic	27
ALS-47	*SYNJ1* c.2771T>G p.Val924Gly	Likely pathogenic	F	None	45	Classic	Unknown
ALS-97	*SYNJ1* c.1655C>T p.Ser552Phe	VUS	F	Dementia	78	Classic	Unknown
ALS-73	*SYNJ1 c.3881C>T* p.Pro1294Leu	VUS	M	None	44	PLMN	44
ALS-88	*SYNJ1 c.3881C>T* p.Pro1294Leu	VUS	F	Dementia	59	Classic	78
ALS-95	*SYNJ1 c.3863C>T* p.Pro1288Leu	VUS	F	None	70	Classic	Unknown
ALS-125	*SYNJ1* c.4266T>A p.Ser1422Arg	VUS	M	None	38	Classic	
ALS-128	*PLA2G6* c.977T>C p.Met326Thr	VUS	F	None	57	Classic	Unknown

Key: ALS, amyotrophic lateral sclerosis; *ATP13A2*, ATPase 13A2; DD, disease duration; *DNAJC6*, DNAJ/HSP40 HOMOLOG, SUBFAMILY C, MEMBER 6; F, female; *FBXO7*, F-box only protein 7; *GCH1*, GTP CYCLOHYDROLASE I; M, male; *PINK1*, PTEN-INDUCED PUTATIVE KINASE 1; *PARK2*, parkin; *PARK7*, PARKINSON DISEASE 7; *PLA2G6*, PHOSPHOLIPASE A2; PLMN; predominant lower motor neuron; PUMN, predominant upper motor neuron; *SYNJ1*, SYNAPTOJANIN 1; VUS, variant of uncertain significance. In bold: patients carrying more than one variant.

**Table 4:** Clinical features in ALS patients carrying *GBA* variants.

Patient	Variant	Classification (Franklin ACMG Classification)	Sex	Family History	Onset Age	Phenotype	DD (Months)
ALS-57	*GBA* c.27 + 5G>C	VUS	F	None	41	Classic	133
ALS-102	*GBA* c.1093G>A p.Glu365Lys + *GBA* c.882T>G p.His294Gln (likely complex allele)	NA	M	None	40	PUMN	Unknown

Key: ALS, amyotrophic lateral sclerosis; DD, disease duration; F, female; *GBA*, ACID β-GLUCOSIDASE; M, male; NA, not available; PUMN, predominant upper motor neuron; VUS, variant of uncertain significance.

**Table 5:** ALS clinical features in patients with at least one PD-related gene variant vs. ALS patients without.

	ALS Patients with at Least One PD-Variant	ALS Patients without PD-Variants	*p*-Value
Onset Age	59.4 (13.4) years *	57.9 (11.7) years *	0.53
Disease Duration (months)	42.5 (39) months **	40 (45) months **	0.93
Family history (ALS/parkinsonism/dementia)	7/30 (23.3%)	21/81 (25.9%)	0.49
ALS Phenotypic variant	Classic 21/30 (70%) PLMN 4/30 (13.3%) PUMN 5/30 (16.7%)	Classic 61/81 (75.3%) PLMN 3/81 (3.7%) PUMN 17/81 (21%)	0.17

* Values (years) are expressed as mean (standard deviation); ** values (months) are expressed as median (interquartile range). ALS cohort after excluding 19 ALS patients carrying a mutation in one of the four major ALS genes. Key: ALS, amyotrophic lateral sclerosis; F, female; M, male; PD, Parkinson’s disease; PLMN, predominant lower motor neuron; PUMN, predominant upper motor neuron.

## Data Availability

All clinical and genetic data are reported in the manuscript and in the supplementary materials.

## Supplementary Materials

The following supporting information can be downloaded at: www.mdpi.com/article/10.3390/genes13081306/s1, Table S1: Genes analyzed in this study; Table S2: Patients carrying at least one variant in the most frequent ALS-related genes; Table S3: Classification of variant in autosomal dominant PD genes in ALS and AD patients; Table S4: Classification of variant in autosomal recessive genes in ALS and AD patients; Table S5: Classification of *GBA* variants in ALS and AD patients.

## References

[1] Phukan J., Elamin M., Bede P., Jordan N., Gallagher L., Byrne S., Lynch C., Pender N., Hardiman O. (2012). The syndrome of cognitive impairment in amyotrophic lateral sclerosis: A population-based study. J. Neurol. Neurosurg. Psychiatry.

[2] Hermosura M.C., Garruto R.M. (2007). TRPM7 and TRPM2-Candidate susceptibility genes for Western Pacific ALS and PD?. Biochim. Biophys. Acta.

[3] Calvo A., Chiò A., Pagani M., Cammarosano S., Dematteis F., Moglia C., Solero L., Manera U., Martone T., Brunetti M. (2019). Parkinsonian traits in amyotrophic lateral sclerosis (ALS): A prospective population-based study. J. Neurol..

[4] Forrest S.L., Kim J.H., De Sousa C., Cheong R., Crockford D.R., Sheedy D., Stevens J., McCrossin T., Tan R.H., McCann H. (2021). Coexisting Lewy body disease and clinical parkinsonism in amyotrophic lateral sclerosis. Eur. J. Neurol..

[5] Van Rheenen W., van der Spek R.A.A., Bakker M.K., van Vugt J.J.F.A., Hop P.J., Zwamborn R.A.J., de Klein N., Westra H.-J., Bakker O.B., Deelen P. (2021). Common and rare variant association analyses in amyotrophic lateral sclerosis identify 15 risk loci with distinct genetic architectures and neuron-specific biology. Nat. Genet..

[6] Giannoccaro M.P., Bartoletti-Stella A., Piras S., Pession A., De Massis P., Oppi F., Stanzani-Maserati M., Pasini E., Baiardi S., Avoni P. (2017). Multiple variants in families with amyotrophic lateral sclerosis and frontotemporal dementia related to C9orf72 repeat expansion: Further observations on their oligogenic nature. J. Neurol..

[7] Bourinaris T., Houlden H. (2018). C9orf72 and its Relevance in Parkinsonism and Movement Disorders: A Comprehensive Review of the Literature. Mov. Disord. Clin. Pract..

[8] Vacchiano V., Mometto N., Bartoletti-Stella A., Rizzo G., Abu-Rumeileh S., Salvi F., Parchi P., Liguori R., Capellari S. (2021). The clinical spectrum of multisystem proteinopathy: Data from a neurodegenerative cohort. J. Neurol. Sci..

[9] Meeus B., Verstraeten A., Crosiers D., Engelborghs S., Broeck M.V.D., Mattheijssens M., Peeters K., Corsmit E., Elinck E., Pickut B. (2012). DLB and PDD: A role for mutations in dementia and Parkinson disease genes?. Neurobiol. Aging.

[10] Vaikath N.N., Erskine D., Morris C.M., Majbour N.K., Vekrellis K., Li J.Y., El-Agnaf O.M.A. (2019). Heterogeneity in α-synuclein subtypes and their expression in cortical brain tissue lysates from Lewy body diseases and Alzheimer’s disease. Neuropathol. Appl. Neurobiol..

[11] Petrucci S., Consoli F., Valente E.M. (2014). Parkinson Disease genetics: A “Continuum” from mendelian to multifactorial inheritance. Curr. Mol. Med..

[12] Riboldi G.M., Frattini E., Monfrini E., Frucht S.J., Di Fonzo A. (2022). A Practical Approach to Early-Onset Parkinsonism. J. Parkinsons Dis..

[13] Cherian A., Divya K.P. (2020). Genetics of Parkinson’s disease. Acta Neurol. Belg..

[14] Sidransky E., Nalls M.A., Aasly J.O., Aharon-Peretz J., Annesi G., Barbosa E.R., Bar-Shira A., Berg D., Bras J., Brice A. (2009). Multicenter analysis of glucocerebrosidase mutations in Parkinson’s disease. N. Engl. J. Med..

[15] Tsuang D., Leverenz J.B., Lopez O.L., Hamilton R.L., Bennett D.A., Schneider J.A., Buchman A.S., Larson E.B., Crane P.K., Kaye J.A. (2012). GBA mutations increase risk for Lewy body disease with and without Alzheimer disease pathology. Neurology.

[16] Canosa A., Grassano M., Moglia C., Iazzolino B., Peotta L., Gallone S., Brunetti M., Barberis M., Sbaiz L., Palumbo F. (2021). GBA variants influence cognitive status in amyotrophic lateral sclerosis. J. Neurol. Neurosurg. Psychiatry.

[17] Bartoletti-Stella A., Baiardi S., Stanzani-Maserati M., Piras S., Caffarra P., Raggi A., Pantieri R., Baldassari S., Caporali L., Abu-Rumeileh S. (2018). Identification of rare genetic variants in Italian patients with dementia by targeted gene sequencing. Neurobiol. Aging.

[18] McKenna A., Hanna M., Banks E., Sivachenko A., Cibulskis K., Kernytsky A., Garimella K., Altshuler D., Gabriel S., Daly M. (2010). The Genome Analysis Toolkit: A MapReduce framework for analyzing next-generation DNA sequencing data. Genome Res..

[19] Bartoletti-Stella A., Vacchiano V., De Pasqua S., Mengozzi G., De Biase D., Bartolomei I., Avoni P., Rizzo G., Parchi P., Donadio V. (2021). Targeted sequencing panels in Italian ALS patients support different etiologies in the ALS/FTD continuum. J. Neurol..

[20] Mighton C., Smith A.C., Mayers J., Tomaszewski R., Taylor S., Hume S., Agatep R., Spriggs E., Feilotter H.E., Semenuk L. (2022). Data sharing to improve concordance in variant interpretation across laboratories: Results from the Canadian Open Genetics Repository. J. Med. Genet..

[21] Kopanos C., Tsiolkas V., Kouris A., Chapple C.E., Albarca Aguilera M., Meyer R., Massouras A. (2019). VarSome: The human genomic variant search engine. Bioinformatics.

[22] Brooks B.R., Miller R.G., Swash M., Munsat T.L., World Federation of Neurology Research Group on Motor Neuron Diseases (2000). El Escorial revisited: Revised criteria for the diagnosis of amyotrophic lateral sclerosis. Amyotroph. Lateral Scler. Other Mot. Neuron Disord..

[23] Chiò A., Moglia C., Canosa A., Manera U., D’Ovidio F., Vasta R., Grassano M., Brunetti M., Barberis M., Corrado L. (2020). ALS phenotype is influenced by age, sex, and genetics: A population-based study. Neurology.

[24] Dubois B., Feldman H.H., Jacova C., Hampel H., Molinuevo J.L., Blennow K., DeKosky S.T., Gauthier S., Selkoe D., Bateman R. (2014). Advancing research diagnostic criteria for Alzheimer’s disease: The IWG-2 criteria. Lancet Neurol..

[25] Birolo G., Aneli S., Di Gaetano C., Cugliari G., Russo A., Allione A., Casalone E., Giorgio E., Paraboschi E.M., Ardissino D. (2021). Functional and clinical implications of genetic structure in 1686 Italian exomes. Hum. Mutat..

[26] Chen Y., Wei Q.Q., Ou R., Cao B., Chen X., Zhao B., Guo X., Yang Y., Chen K., Wu Y. (2015). Genetic Variants of SNCA Are Associated with Susceptibility to Parkinson’s Disease but Not Amyotrophic Lateral Sclerosis or Multiple System Atrophy in a Chinese Population. PLoS ONE.

[27] Guo X.Y., Chen Y.P., Song W., Zhao B., Cao B., Wei Q.Q., Ou R.W., Yang Y., Yuan L.X., Shang H.-F. (2014). SNCA variants rs2736990 and rs356220 as risk factors for Parkinson’s disease but not for amyotrophic lateral sclerosis and multiple system atrophy in a Chinese population. Neurobiol. Aging.

[28] Da Silva C.P., de MAbreu G., Cabello Acero P.H., Campos MJúnior Pereira J.S., de ARamos S.R., Nascimento C.M., Voigt D.D., Rosso A.L., Araujo Leite M.A., Vasconcellos L.F.R. (2017). Clinical profiles associated with LRRK2 and GBA mutations in Brazilians with Parkinson’s disease. J. Neurol. Sci..

[29] Wszolek Z.K., Vieregge P., Uitti R.J., Gasser T., Yasuhara O., McGeer P., Berry K., Calne D.B., Vingerhoets F.J.G., Klein C. (1997). German Canadian family (family A) with parkinsonism, amyotrophy, and dementia: Longitudinal observations. Parkinsonism Relat. Disord..

[30] Whittle A.J., Ross O.A., Naini A., Gordon P., Mistumoto H., Dächsel J.C., Stone J.T., Wszolek Z.K., Farrer M.J., Przedborski S. (2007). Pathogenic Lrrk2 substitutions and Amyotrophic lateral sclerosis. J. Neural. Transm..

[31] Ciani M., Bonvicini C., Scassellati C., Carrara M., Maj C., Fostinelli S., Binetti G., Ghidoni R., Benussi L. (2019). The Missing Heritability of Sporadic Frontotemporal Dementia: New Insights from Rare Variants in Neurodegenerative Candidate Genes. Int. J. Mol. Sci..

[32] Caesar M., Felk S., Zach S., Brønstad G., Aasly J.O., Gasser T., Gillardon F. (2014). Changes in matrix metalloprotease activity and progranulin levels may contribute to the pathophysiological function of mutant leucine-rich repeat kinase 2. Glia.

[33] Ho D.H., Kim H., Nam D., Sim H., Kim J., Kim H.G., Son I., Seol W. (2018). LRRK2 impairs autophagy by mediating phosphorylation of leucyl-tRNA synthetase. Cell Biochem. Funct..

[34] Nuytemans K., Theuns J., Cruts M., Van Broeckhoven C. (2010). Genetic etiology of Parkinson disease associated with mutations in the SNCA, PARK2, PINK1, PARK7, and LRRK2 genes: A mutation update. Hum. Mutat..

[35] Gaweda-Walerych K., Sitek E.J., Narożańska E., Buratti E. (2021). Parkin beyond Parkinson’s Disease-A Functional Meaning of Parkin Downregulation in TDP-43 Proteinopathies. Cells.

[36] Dilliott A.A., Abdelhady A., Sunderland K.M., Farhan S.M.K., Abrahao A., Binns M.A., Black S.E., Borrie M., Casaubon L.K., Dowlatshahi D. (2021). Contribution of rare variant associations to neurodegenerative disease presentation. NPJ Genom. Med..

[37] Lagier-Tourenne C., Polymenidou M., Hutt K.R., Vu A.Q., Baughn M., Huelga S.C., Clutario K.M., Ling S.C., Liang T.Y., Mazur C. (2012). Divergent roles of ALS-linked proteins FUS/TLS and TDP-43 intersect in processing long pre-mRNAs. Nat. Neurosci..

[38] Knippenberg S., Sipos J., Thau-Habermann N., Körner S., Rath K.J., Dengler R., Petri S. (2013). Altered expression of DJ-1 and PINK1 in sporadic ALS and in the SOD1(G93A) ALS mouse model. J. Neuropathol. Exp. Neurol..

[39] Schapira A.H., Chiasserini D., Beccari T., Parnetti L. (2016). Glucocerebrosidase in Parkinson’s disease: Insights into pathogenesis and prospects for treatment. Mov. Disord..

[40] Guerreiro R., Ross O.A., Kun-Rodrigues C., Hernandez D.G., Orme T., Eicher J.D., Shepherd C.E., Parkkinen L., Darwent L., Heckman M.G. (2018). Investigating the genetic architecture of dementia with Lewy bodies: A two-stage genome-wide association study. Lancet Neurol..

[41] Huang Y., Deng L., Zhong Y., Yi M. (2018). The Association between E326K of *GBA* and the Risk of Parkinson’s Disease. Parkinsons Dis..

[42] Kim H.M., Nazor C., Zabetian C.P., Quinn J.F., Chung K.A., Hiller A.L., Hu S.-C., Leverenz J.B., Montine T.J., Edwards K.L. (2019). Prediction of cognitive progression in Parkinson’s disease using three cognitive screening measures. Clin. Park Relat. Disord..

[43] Oliveira L.M., Rastin T., Nimmo G.A.M., Ross J.P., Dion P.A., Zhang M., Nevay D.L., Arkadir D., Gotkine M., Barnett C. (2021). Occurrence of Amyotrophic Lateral Sclerosis in Type 1 Gaucher Disease. Neurol. Genet..

