# Supplementary material for: Frequency of Parkinson’s Disease Genes and Role of PARK2 in Amyotrophic Lateral Sclerosis: An NGS Study
Source: Genes (Basel). 2022 Jul 22;13(8):1306. doi: 10.3390/genes13081306 (PMC9332209; doi:10.3390/genes13081306)
Supplement: Supplementary file 1 [file genes-13-01306-s001.zip › genes-1768203-supplementary.pdf]

Frequency of Parkinson's Disease genes and role of *PARK2* in Amyotrophic Lateral Sclerosis: a NGS study

Veria Vacchiano[1,2], Anna Bartoletti-Stella[3], Giovanni Rizzo[1], Patrizia Avoni[1,2], Piero Parchi[1,2], Fabrizio Salvi[1], Rocco Liguori[1,2], Sabina Capellari[1,2]

[1]IRCCS Istituto delle Scienze Neurologiche di Bologna, Bellaria Hospital, 40139 Bologna, Italy.

[2]Dipartimento di Scienze Biomediche e Neuromotorie (DIBINEM), Università di Bologna, 40123 Bologna, Italy

[3]Department of Experimental Diagnostic and Specialty Medicine (DIMES), University of Bologna, 40138 Bologna, Italy*

Correspondence: sabina.capellari@unibo.it

Supplementary Materials

Table S1: Genes analyzed in this study

Symbol	Name	PD Role [OMIM]	Inheritance
GBA	ACID BETA-GLUCOSIDASE	Lewy body dementia, susceptibility to; Parkinson disease, late-onset, susceptibility to	AD; multifactorial
SNCA	SYNUCLEIN, ALPHA	Lewy body dementia; Parkinson disease 1; Parkinson disease 4	AD
LRRK2	LEUCINE-RICH REPEAT KINASE 2	Parkinson disease 8	AD
GCH1	GTP CYCLOHYDROLASE I	Dystonia, DOPA-responsive, with or without hyperphenylalaninemia	AD, AR
PINK1	PTEN-INDUCED PUTATIVE KINASE 1	Parkinson disease 6, early onset	AR
PARK2	PARKIN	Parkinson disease, juvenile, type 2	AR
PARK7	PARKINSON DISEASE 7	Parkinson disease 7, autosomal recessive early-onset	AR
SYNJ1	SYNAPTOJANIN 1	Parkinson disease 20, early-onset	AR
PLA2G6	PHOSPHOLIPASE A2	Parkinson disease 14, autosomal recessive	AR
ATP13A2	ATPase 13A2	Kufor-Rakeb syndrome	AR
DNAJC6	DNAJ/HSP40 HOMOLOG, SUBFAMILY C, MEMBER 6	Parkinson disease 19a, juvenile-onset; Parkinson disease 19b, early-onset	AR
FXBO7	F-box only protein 7	Parkinson disease 25, early onset	AR

Key: AD, autosomal dominant; AR, autosomal recessive.

Table S2: Patients carrying at least one variant in the most frequent ALS-related genes

Gene	ALS Patients N (%)
C9ORF72	8 (6.2)
SOD1	5 (3.8)
FUS	2 (1.5)
TARBP	4 (3.1)
Total	19 (14.6)

Key: ALS, amyotrophic lateral sclerosis

Table S3: Classification of variant in autosomal dominant PD genes in ALS and AD patients

Patient	VARIANT	GNOMAD EU	Franklin ACMG Classification	Other mutated gene
**ALS-25**	***LRRK2*** **c.7470A>C p.Gln2490His**	**NR**	**VUS (PM2, BP4)**	***PARK2***
ALS-29	*LRRK2* c.7315C>A p.Leu2439Ile	0.00004408	VUS (PM2, BP4)	
**AD-36**	***LRRK2*** **c.6783T>A p.Asn2261Lys**	**NR**	**VUS (PM2, BP4)**	***ATP13A2***
AD-47	*LRRK2* c.683G>C p.Cys228Ser	0.0001632	VUS (PM2, BP4)	

Key: ACMG, American College of Medical Genetics and Genomics guidelines for the interpretation of sequence variants provided by Franklin-Genoox (for details see Materials and Methods section); AD, Alzheimer's disease; ALS, amyotrophic lateral sclerosis; BP4, benign supporting; NR, not reported; VUS, variant of uncertain significance; PM2, pathogenic moderate. In bold patients carrying more than one variant. For reasons of simplification and data readability, we only report patients with at least one variant in Parkinson's Disease causative genes. Patients with no variants were not detailed in the Table.

Table S4: Classification of variant in autosomal recessive genes in ALS patients

Patient	VARIANT	GNOMAD EU	Franklin ACMG Classification	Other mutated gene
ALS-24	*ATP13A2* c.2836A>T p.Ile946Phe	0.001549	VUS (PM2)	
ALS-40	*ATP13A2* c.1382delC p.Ala461ValfsTer5	NR	Likely pathogenic (PVS1, PM2)	
**ALS-46**	***ATP13A2*** **c.3059A>G p.Tyr1020Cys**	**0.00004738**	**VUS (PM2)**	***SOD1***
ALS-91	*ATP13A2* c.2947C>T p.Pro983Ser	0.00001787	VUS (PM2, PP3)	
ALS-2	*CHCHD2* c.101C>T p.Pro34Leu	0.0002105	VUS (PM2)	
ALS-2	*DNAJC6* c.1112A>C p.Lys371Thr	NR	VUS (PP3, PM2)	
ALS-27	*DNAJC6* c.829G>A p.Ala277Thr	0.0008239	VUS (BS1)	
ALS-81	*DNAJC6* c.2048C>T p.Thr683Met	0.00003519	VUS (PM2)	
ALS-60	*FBXO7* c.301A>C p.Asn101His	0.000008794	VUS (PM2, BP4)	
**ALS-98**	***FBXO7*** **c.1538G>A p.Arg513Gln**	**NR**	**VUS (PM2)**	***PINK1***
ALS-15	*PINK1* c.1342G>A p.Gly448Arg	0.00004403	VUS (PM2, PP3)	
ALS-18	*PINK1* c.434C>T p.Thr145Met	0.00004396	VUS (PM2, BP4)	
ALS-53	*PINK1* c.1609G>A p.Ala537Thr	0.00003097	VUS (PM2)	
**ALS-98**	***PINK1*** **c.587C>T p.Pro196Leu**	0.0003176	VUS (PM2)	***FBXO7***
ALS-106	*PINK1* c.587C>T p.Pro196Leu	0.0003176	VUS (PM2)	
ALS-103	*PARK7* c.535G>A p.Ala179Thr	0.0009910	VUS (BP4)	

ALS-28	*GCH1*<br>c.671A>G p.Lys224Arg	0.000513	Likely pathogenic (PP5, PM2, PM1, PP2)	
ALS-12	*PARK2*<br>c.1204C>T p.Arg402Cys	0.002245	VUS (BS2)	
**ALS-25**	***PARK2***<br>**c.1204C>T p.Arg402Cys**	**0.002245**	VUS (BS2)	***LRRK2***
ALS-64	*PARK2*<br>c.1204C>T p.Arg402Cys	0.002245	VUS (BS2)	
ALS-80	*PARK2*<br>c.1204C>T p.Arg402Cys	0.002245	VUS (BS2)	
ALS-100	*PARK2*<br>c.701G>A p.Arg234Gln	0.0002940	VUS (PM2)	
ALS-113	*PARK2*<br>c.1204C>T p.Arg402Cys	0.002245	VUS (BS2)	
ALS-126	*PARK2*<br>c.1204C>T p.Arg402Cys	0.002245	VUS (BS2)	
ALS-129	*PARK2*<br>c.436T>C p.Phe146Leu	NR	VUS (PP3, PM2)	
ALS-47	*SYNJ1*<br>c.2771T>G p.Val924Gly	NR	Likely pathogenic (PP3, PM2)	
ALS-73	*SYNJ1*<br>c.3881C>T p.Pro1294Leu	0.0001497	VUS (PM2)	
ALS-88	*SYNJ1*<br>c.3881C>T p.Pro1294Leu	0.0001471	VUS (PM2)	
ALS-95	*SYNJ1*<br>c.3863C>T p.Pro1288Leu	0.0003329	VUS (BS2)	
ALS-97	*SYNJ1*<br>c.1655C>T p.Ser552Phe	NR	VUS (PM2, PP3)	
ALS-125	*SYNJ1*<br>c.4266T>A p.Ser1422Arg	NR	VUS (PM2)	
ALS-128	*PLA2G6*<br>c.977T>C p.Met326Thr	0.00002895	VUS (PM2, PM1, PP2)	
AD-19	*ATP13A2*<br>c.1537G>A p.Asp513Asn	NR	Likely pathogenic (PP3, PM2)	
**AD-36**	***ATP13A2***<br>**c.3320C>T p.Ala1107Val**	**0.00002398**	**VUS (PM2, BP4)**	***LRRK2***
**AD-38**	***ATP13A2***<br>**c.2062G>A p.Val688Met**	**NR**	**VUS (PM2)**	***PLA2G6***
AD-55	*ATP13A2*<br>c.1630C>T p.Arg544Cys	0.00007721	VUS (PM2, BP4)	
AD-57	*CHCHD2*<br>c.134C>G p.Ser45Cys	NR	VUS (PM2, BP4)	
AD-71	*DNAJC6*<br>c.397A>T p.Met133Leu	0.0006365	VUS (PP3, BS1)	
**AD-20**	***FBXO7***<br>**c.277T>A p.Ser93Thr**	**NR**	**VUS (PM2, BP4)**	***PARK2***
AD-74	*PARK2*<br>c.1028C>T p.Pro343Leu	0.0001087	VUS (PM2, PP3)	
**AD-20**	***PARK2***<br>**c.719C>T p.Thr240Met**	**NR**	**Pathogenic (PP5, PM2, PP3, PM5)**	***FBXO7***
AD-52	*PARK2*<br>c.719C>T p.Thr240Met	0.0002323	Pathogenic (PP5, PM2, PP3, PM5)	
AD-56	*PARK2*<br>c.455G>T p.Gly152Val	NR	VUS (PM2)	
AD-15	*PINK1*<br>c.1600_1602dupCAA<br>p.Gln534dup	0.000008790	VUS (PM2, PM4, PP5)	
**AD-38**	***PLA2G6***<br>**c.1426A>G p.Thr476Ala**	**0.00003878**	**VUS (PM2, PP2)**	***ATP13A2***
AD-61	*PLA2G6*<br>c.284A>G p.Tyr95Cys	NR	VUS (PM2, PP2)	
**AD-14**	***SYNJ1***<br>**c.3533G>A p.Arg1178Lys**	**NR**	**VUS (PM2)**	***GBA***
AD-45	*SYNJ1*<br>c.4266T>A p.Ser1422Arg	**0.00001551**	VUS (PM2)	
AD-50	*SYNJ1*<br>c.1720G>A p.Val574Ile	0.0001785	VUS (PM2)	
AD-87	*SYNJ1*<br>c.3863C>T p.Pro1288Leu	0.0003329	VUS (PM2)	

Key: ACMG, American College of Medical Genetics and Genomics guidelines for the interpretation of sequence variants provided by Franklin-Genoox (for details see Materials and Methods section); AD, Alzheimer's disease; ALS, amyotrophic lateral sclerosis; BS1 and BS2, benign strong; BP4, benign supporting; NR, not reported; PM1, PM2, PM4 and PM5, pathogenic moderate; PP2, PP3 and PP5, pathogenic supporting; PVS1, pathogenic very strong; VUS, variant

of uncertain significance. In bold patients carrying more than one variant. For reasons of simplification and data readability, we only report patients with at least one variant in Parkinson's Disease causative genes. Patients with no variants were not detailed in the Table.

Table S5: Classification of *GBA* variants in ALS and AD patients

Patient	VARIANT	GNOMAD EU	Franklin ACMG Classification	Other mutated gene
ALS-57	GBA<br>c.27+5G>C	NR	VUS (PM2)	
ALS-102	*GBA*<br>c.1093G>A p.Glu365Lys<br><br>*GBA*<br>c.882T>G p.His294Gln	NR	NA, Likely complex allele	
AD-1	*GBA*<br>c.882T>G p.His294Gln	0.0003025	Likely pathogenic (PM2, PM1, PP2, PP5)	
**AD-14**	***GBA***<br>**c.882T>G p.His294Gln**	**0.0003025**	**Likely pathogenic (PM2, PM1, PP2, PP5)**	***SYNJ1***
AD-59	*GBA*<br>c.1224G>A p.Thr408=	0.0003103	VUS (PM2, PP3)	

Key: ACMG, American College of Medical Genetics and Genomics guidelines for the interpretation of sequence variants provided by Franklin-Genoox (for details see Materials and Methods section); AD, Alzheimer's disease; ALS, amyotrophic lateral sclerosis; NR, not reported; PM1 and PM2, pathogenic moderate; PP2, PP3 and PP5, pathogenic supporting; VUS, variant of uncertain significance. In bold patients carrying more than one variant. For reasons of simplification and data readability, we only report patients with at least one variant in Parkinson's Disease causative genes. Patients with no variants were not detailed in the Table.
